# Pharmacokinetics Education: Addressing Core Learning Challenges Through Innovations in Teaching

**DOI:** 10.1002/prp2.70286

**Published:** 2026-07-04

**Authors:** Catherine Dobler, Jiani Wu, Grace Leaman, Leah Brassard, Michelle Arnot, Fatima Mraiche

**Affiliations:** ^1^ Department of Pharmacology, Faculty of Medicine and Dentistry, College of Health Sciences University of Alberta Edmonton Alberta Canada; ^2^ Department of Pharmacology and Toxicology, Temerty Faculty of Medicine University of Toronto Toronto Ontario Canada

## Abstract

Pharmacokinetics is fundamental to drug discovery, therapeutic optimization, and patient safety. Despite its importance, students consistently demonstrate limited mastery of pharmacokinetic core concepts. Studies reveal that students have persistent misconceptions of pharmacokinetic core concepts, difficulty applying mathematical principles, and challenges in transferring theoretical knowledge to medical and biomedical practices. Contributing factors include mathematical skill deficits upon entering university programs, fragmented curricular integration, and limited opportunities for active application of knowledge. These gaps raise concerns about the preparedness of graduates entering health professions and drug design/discovery fields, where pharmacokinetic competence directly impacts the fundamentals of drug discovery, therapeutic optimization, and patient safety. In addition to student challenges with learning pharmacokinetics, this commentary highlights innovative teaching strategies for educators to support student comprehension and application of pharmacokinetic core concepts. Approaches such as team‐based learning, case‐based exercises, simulations, games, and prerequisite review tutorials have shown promise in strengthening conceptual understanding and bridging theory with practice. These strategies have largely been studied in isolated contexts, underscoring the need for broader evaluations examining how course design, delivery methods, and teaching strategies shape student learning of pharmacokinetic core concepts. Future research could explore pharmacology educator and student perspectives on pharmacokinetics to develop an international framework for pharmacokinetics education. Such a framework would ensure progressive reinforcement of core concepts, integration across curricula, and alignment of teaching practices with real‐world application, ultimately preparing students for diverse careers where pharmacokinetics proficiency is essential.

## Graphical abstract

Student learning of pharmacokinetic core concepts is challenged by mathematical complexity, abstract concepts and misconceptions, and difficulty translating theory into practice. Educational strategies, including active and case‐based learning, simulation and visualization, and curriculum integration, may strengthen understanding and application of the pharmacokinetic core concepts of absorption, distribution, metabolism, and excretion (ADME), thereby improving pharmacokinetic competence and supporting drug discovery, therapeutic optimization, and patient safety.

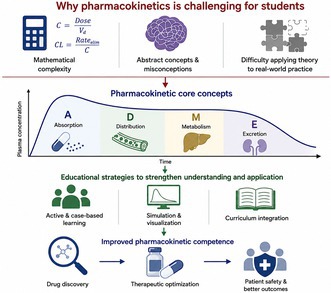

Pharmacology is an ever‐evolving field, shaped not only by research discoveries and clinical innovations, but also by the pedagogical innovations in pharmacology education. Pharmacology education is integral to various academic programs including life sciences and allied health professions [1]. Efforts, such as those led by White et al. [2], have sought to define core concepts foundational to pharmacology education through an international, collaborative approach. These concepts are categorized into three domains: pharmacodynamics, pharmacokinetics, and patient outcomes. Pharmacokinetic core concepts, such as absorption, bioavailability, distribution, metabolism, and elimination, form the foundation for understanding drug behavior [2]. These concepts remain difficult for students to learn and master [3, 4, 5]. Consequently, scholars have sought to improve teaching practices for pharmacokinetics to address student challenges [6, 7, 8, 9, 10, 11]. This commentary examines why learning pharmacokinetic core concepts is challenging for students and outlines innovative teaching strategies aimed at improving both student comprehension and practical application of core concepts.

## 1 Challenges in Learning Pharmacokinetics Core Concepts

Although pharmacokinetics plays a critical role in understanding drug behavior, it remains a challenging area for students to grasp. Second year health and life sciences students consistently performed worse on pharmacokinetic‐related exam questions compared to those assessing pharmacodynamics concepts [3]. Pandit et al., analyzed medical student exam questions and found that students frequently failed pharmacokinetic sections [4]. However, since these questions comprised only a small portion, most students passed the exam overall, despite limited understanding of pharmacokinetic concepts. Furthermore, a survey of medical students assessing self‐rated knowledge and skills in pharmacokinetics revealed that students did not perceive an improvement in pharmacokinetic understanding as their program advanced [12]. Additionally, Babey et al., evaluated students from diverse disciplines requiring pharmacokinetic knowledge to understand their grasp of core concepts by examining their ability to explain and apply four key principles: drug bioavailability, drug clearance, volume of distribution, and steady‐state concentration [5]. Their findings showed that students rarely provided definitions of core concepts that fully aligned with expert definitions, with most omitting at least one essential component per concept. The authors noted that this difficulty in defining pharmacokinetic core concepts may contribute to student challenges in applying pharmacokinetic principles in real‐world scenarios.

To fully address the gaps in student learning of pharmacokinetics, it is important to identify the underlying reasons for students' difficulties. Pharmacokinetics relies heavily on mathematics, a subject that many students find intimidating due to limited prior exposure [13]. Limited mathematical proficiency may hinder students' ability to apply pharmacokinetics in real‐world contexts. In an exam given to first‐year bioscience students, many struggled with mathematical word problems contextualized in biology [14]. This challenge may be mirrored among pharmacology students, for whom such mathematical problems are critical to applying pharmacokinetic principles in practice. Addressing student deficits in core mathematics education and bridging gaps between student comprehension and application to real‐world practice may be critical targets for educators to improve student learning of pharmacokinetics.

## 2 Challenges in Teaching Pharmacokinetics Core Concepts

A 2016 survey of U.S. pharmacy schools reported that approximately 41% of programs had shifted from offering clinical pharmacokinetics as a stand‐alone course to integrating it throughout program curricula [6]. This is advantageous because when concepts are addressed at several stages throughout a program, it allows for reinforcement and advancement of concepts. The survey also identified that approximately one in three surveyed pharmacy programs do not teach basic pharmacokinetic principles prior to clinical pharmacokinetics courses. Thus, it is unsurprising that students struggle to apply pharmacokinetic principles in clinical settings without a foundation of basic pharmacokinetic concepts. Addressing inconsistencies in how pharmacokinetics is integrated into curricula requires a comprehensive approach that includes restructuring curricula to provide stronger foundational knowledge (particularly with mathematics), reinforcing core concepts across multiple years, and incorporating active learning strategies to bridge theory and clinical application. As pharmacokinetics relies on the capacity to apply abstract theoretical principles to real‐world contexts, explicit instructions on how to translate core concepts to clinical applications, such as through simulations of real‐world scenarios, may be beneficial for students [7]. A graded approach to pharmacokinetics instruction that establishes essential background knowledge and progressively reinforces and advances concepts across successive program years may benefit students.

## 3 Innovative Teaching Strategies for Learning Pharmacokinetic Core Concepts

**FIGURE 1:**
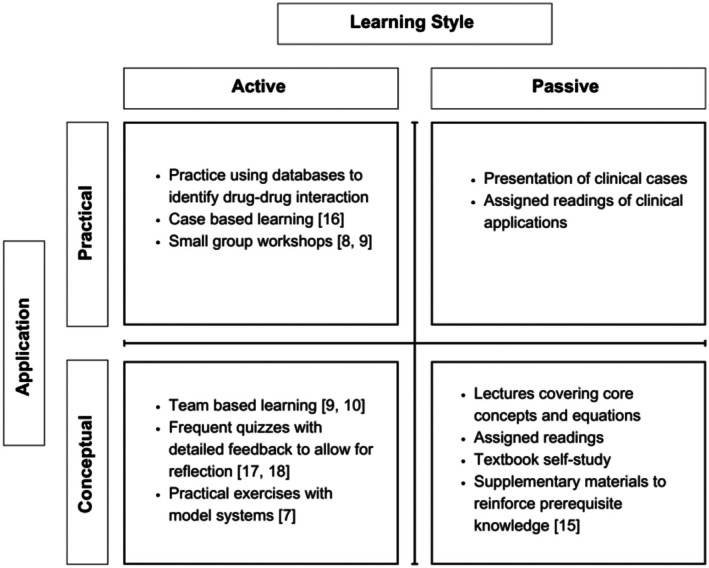
Categorization of learning strategies for pharmacokinetic education based on active or passive learning styles and their application to fostering practical or conceptual knowledge. The strategies presented here represent various methods historically used to teach pharmacokinetics, including passive approaches like lectures, which focus on conceptual learning. In addition to the traditional teaching methods, strategies have been developed to enhance existing pedagogical methods and improve student understanding, such as active learning techniques like case‐based learning.

In response to the challenges students face in learning pharmacokinetics core concepts, educators have explored a range of innovative teaching strategies designed to improve student engagement, comprehension, and application of core concepts (Table 1). To date, successful revisions of pharmacokinetics teaching have included small‐group workshops that promote active participation and build student confidence in applying concepts clinically [8], as well as increased use of active learning strategies such as team‐based learning [9, 10]. Another impactful technique includes hands on simulators and practical exercises using model systems to reinforce principles of compartmental modeling and other clinically relevant pharmacokinetic factors such as volume of distribution [7]. Incorporating games to teach and review pharmacokinetic core concepts fostered critical thinking and teamwork while also demonstrating a positive effect on student performance in pharmacokinetics assessments [11]. Small group workshops improved students' confidence in their ability to apply pharmacokinetic theory and allowed them to feel as though they were involved in an active learning process [8]. Using online prerequisite review tutorials in subjects such as biology, chemistry, and physiology helped to identify foundational topics that students may need to improve and prepared pharmacy students for their future coursework [15]. Other attempts to improve student learning include formative feedback prior to heavier weighted assessments [17] and providing more frequent assessment [18] as opportunities for students to learn and reflect on content. Case‐based learning with small groups enhanced students' self‐assessed ability to apply pharmacokinetic core concepts and improved test scores [16]. These different approaches include both passive and active teaching techniques and have been employed to improve conceptual or practical pharmacokinetic knowledge as they would be used in clinical practice (Figure 1). These innovative approaches (summarized in Table 1) demonstrate the potential for teaching strategies to enhance student outcomes by bridging the gap between theoretical knowledge and practical application. Given that each of these teaching approaches has been evaluated in a single context, comprehensive large‐scale studies may be needed to confirm their efficacy across varied educational settings.

**TABLE 1:** Challenges in learning pharmacokinetics and teaching strategies to support students.

Challenge	Teaching strategies
Difficulty grasping pharmacokinetic core concepts relative to pharmacodynamic concepts	Greater cohesion across academic years to ensure progressive development of concepts and prevent key topics from being overlooked [6] Provide students with Supporting Information to strengthen pre‐requisite knowledge vital to understanding pharmacokinetic concepts (such as necessary knowledge in chemistry or math) [15]
Difficulty translating knowledge into real world application	Hands‐on simulators or practical exercises to demonstrate concepts [7] Active learning approaches such as case‐based learning with clinically applicable scenarios to practice applying concepts [9, 16]
Difficulty applying mathematical concepts and compartmental modeling	Seminars or small group learning to provide opportunities to practice using formulas [8, 9] Frequent quizzes with detailed feedback to facilitate students identifying areas of weaknesses and allowing time to improve understanding [17, 18]

## 4 Future Directions for Teaching Pharmacokinetics

Although there are a number of studies on student difficulties in learning pharmacokinetics core concepts in the context of health professional programs such as pharmacy and medical education [4, 6, 19], the equivalent is lacking in undergraduate pharmacology and biomedical programs. A strong foundation in pharmacokinetics is critical even at the undergraduate level, as many students go on to allied health professions or the drug development industry, where understanding pharmacokinetics results in more efficient drug development and better patient outcomes. Given the lack of literature on undergraduate pharmacokinetics education, future research should seek to address this gap by examining how teaching interventions shape student learning in pharmacokinetics. Additionally, broad scholarship in this area may contribute to a deeper understanding of how different approaches to teaching pharmacokinetic core concepts perform across varied contexts. Revisiting pharmacokinetic principles throughout an undergraduate program could serve to enhance student understanding rather than a “one and done” course approach that is often modular and insulated. Involving students in the process of refining teaching strategies is another avenue for future research, as the students are the primary beneficiaries of such improvements. Student perspectives that are gathered through surveys or focus groups will be vital for developing and integrating effective and successful teaching approaches. Developing an international framework that builds on the core competencies developed by White and colleagues ensures students acquire essential prerequisite knowledge and engage with pharmacokinetics in a structured, progressive manner that supports concept mastery as pharmacokinetics competence is crucial for students exploring diverse career paths in allied health professions and drug discovery field [2]. Finally, pharmacokinetic pedagogy must evolve to incorporate generative AI literacy and competency development to prepare students for a rapidly changing healthcare and pharmaceutical landscape [20]. Generative AI will influence areas such as drug discovery and development, as well as personalized medicine; educators should incorporate pedagogical innovation that integrates AI literacy to equip students with the critical thinking and evaluative skills necessary to effectively and responsibly engage with these emerging technologies in future professional practice.

## 5 Conclusion

Student gaps in learning pharmacokinetics core concepts have been suggested to stem from areas such as the mathematical basis of pharmacokinetics, deficits in the ability to translate theoretical knowledge to clinical practice, and a lack of cohesion in curriculum structure across programs [6, 8, 13]. While it is apparent that students are struggling with a deep understanding of pharmacokinetics core concepts, the need for improved teaching strategies to combat their challenges is being recognized and improved upon through various active learning approaches and reinforcement of prerequisite knowledge [8, 9, 15, 16]. As more pharmacology educators identify these struggles, scholars have started to explore these teaching strategies and educator perspectives in undergraduate programs in order to develop an international framework that supports student learning of pharmacokinetics.

## Author Contributions

F.M. and M.A. conceived the article outline, developed the perspective outline, and contributed to drafting and revising the manuscript. C.D. and J.W. contributed to writing and revising the manuscript. G.L. and L.B. provided revisions and feedback on the manuscript. All authors reviewed and approved the final version of the manuscript.

## Conflicts of Interest

The authors declare no conflicts of interest.

## Data Availability

Data sharing is not applicable to this article as no datasets were generated or analysed during the current study.
